# The Medical, Surgical, and Hygienic Exhibition

**Published:** 1899-05-27

**Authors:** 


### THE MEDICAL, SURGICAL, AND HYGIENIC EXHIBITION.

The Medical, Surgical, and Hygienic Exhibition which was opened last Tuesday at the Queen's Hall bids fair to become an annual institution.

The exhibitions held in previous years have been attended by large numbers, and, we believe, have been regarded by exhibitors as very successful, and if we may judge by the numbers present on the opening day and by the representative character of the exhibits, the present one is likely to surpass even its predecessors. The nature of the show is very much like that to which visitors to the annual meetings of the British Medical Association have long been accustomed; in fact, we believe that this series of exhibitions came into being on the occasion when the British Medical Association met in Canada. This was too far for the exhibitors to follow, so they set up a show of their own, which has been repeated annually since. To the practitioner the chief interest will probably be divided between the really splendid show of surgical instruments and dressings and the array of invalid and other dietetic preparations. Mere drugs are not, perhaps, so prominent as is sometimes the case, but some of the best known and most eminent firms are well represented. Nurses will find much to interest them, partly in the appliances made specially for their use, partly in the foods and dressings and instruments which are present in such profusion on every side.

To commence at the very beginning, and to take milk as the type of a food, it is interesting to note what a firm hold the preservation of milk by simple sterilisation has taken among the food industries of the country. The Aylesbury Dairy Company have a fine exhibit of specimens illustrating the researches carried out in the company's laboratory, and show samples of humanised milk, peptonised milk, whey, and ordinary milk and cream, all perfectly sterilised. They also show various modifications of milk for special purposes, and of koumiss in four different varieties.

The "Excel" Milk Sterilising Company show sterilised milk and sterilised cream in bottles, and also sterilised milk in tins, the latter chiefly intended for export and for use in places from which it would not be convenient to return the bottles. The Friern Manor Farm have sterilised milk, and milk humanised by Dr. Gaertner's process, and Messrs. Welford and Sons show humanised milk, sterilised milk, asses milk, koumiss, &c.

But great efforts seem to be now made to deliver milk fresh, unsterilised, und untampered with, Messrs. Welford undertaking to deliver their nursery milk in London within two hours of milking, and the Aylesbury Company being able, by careful attention to details, to distribute milk in the metropolis from their country farms free from any preservative and unsterilised.

Many specimens of condensed milk were also shown, among which may be mentioned the Viking brand, by Henri Nestlé.

Turning to other food preparations we find a large array suitable for a great variety of conditions. G. Van Abbott and Sons have a good display of foods for diabetics, and of the various materials used in their preparation. Gluten bread, soya bread, bran gluten bread, almond bread, &c., are shown, as well as the same materials made up in biscuits, cakes, and other tempting forms, together with other forms of prepared food suitable for diabetic patients.

Messrs. Callard again show foods for diabetics and for those who have to avoid starch in their diet. Not only are the more ordinary forms of gluten preparation to be seen, but some really tasty confectionery. The sponge cakes were especially worthy of recommendation, and the iodine solution showed them to be free from starch.

In regard to meat preparations, we can but mention V. Benoist, with his clear turtle soup; the Bovril Company, with their well-known preparations put up in an infinite variety of forms; Stephen Smith and Co., with their Keystone Beef Wine; the Liebig's Extract of Meat Company's preparation; Brand and Co.'s well-known and most palatable essences of beef, chicken, &c.; and G. Nelson Dale and Co., with "Hipi" pure mutton essence. Nor should we omit to notice in connection with foods the very useful series of digestive substances prepared by Fairchild Brothers and Foster—Pepsin, Peptogenic Milk Powder, "Zymine" Peptonising tubes, &c.

Malt extracts were represented by the Maltine Manufacturing Company, who showed many combinations of malt with various medicinal and dietetic substances; by M. and Leopold Hoff, who contented themselves with showing their well-known and long recognised malt extract; and by Price's, who showed their "Glycerol of Malt." Among alimentary substances may also be placed Scott's Emulsion, shown by Scott and Bowne, although no doubt its therapeutic utility might lead one with equal justice to place it among the drugs. Among dietetic articles should also be mentioned Fromm's Vegetable Extract and Soup Tablets, Cadbury's Cocoa, and the Mosquera's Beef Jelly and various nutrient suppositories shown by Parke, Davis, and Co.

Among tea-pots, that sold by the Bella-Wattee Company seems to deserve mention as one of the best, if not actually the best, of the various methods of not only brewing good tea which is a comparatively simple affair, but of getting a good second cup—a much more difficult matter.

Mineral and aërated waters were well represented. The Kronthal Company with their three kinds of waters; Ingram and Royle with Vichy, Carlsbad, and Hunyadi Janos waters; S. Kutnow and Co. with their Effervescing Carlsbad Powders; Alexander Riddle and Co. with Stower's Lime Juice Cordial and Lemon Squash; Davy, Hill, Son, Yates, and Hicks with specimens of Esvach Water; and Cooper and Co. with their "Oxy-carbonated" waters were all ready to demonstrate the virtues of their preparations.

Plenty of belts and corsets were on view. The "Domen" belts seem sensibly planned.

India-rubber goods were to be met with on various stalls. We were much struck with the advantages of the "Paralytic Combination Water-bed and Sheet" shown by Messrs. Anderson, Anderson, and Anderson.

Among the pharmaceutical chemists who exhibited drugs and other preparations may be mentioned W. Martindale; Oppenheimer, Son, and Co.; Cooper and Co.; and Macfarlan and Co.

Coming to the more purely surgical side of the exhibition we find the instrument makers very well represented.

John Weiss and Son show a large number of their well-known instruments. It is impossible to refer to these in detail, but we would mention Déchery's Automatic Cautery, a great advance over the old style of thermo-cautery, and also the display of lithotrites and bladder aspirators. An all-metal syringe is a great improvement. It has no packing whatever, and works quite smoothly.

Mayer and Meltzer, among other things, have a good display of anæsthetic inhalers according to the methods of various administrators. Attention may be drawn to their New Aseptic Clover's Inhaler, with little windows, or "man-holes," through which the interior can be cleaned. Their University Tourniquet is an improvement on the ordinary forms.

Down Bros. have a full display of modern surgical instruments, among which we noticed Tubby's Talipes Wrench, and sets of instruments for the screwing and suturing of bone, as recommended by Arbuthnot Lane.

Evans and Wormull show surgical instruments of many patterns.

Wm. K. Stacy shows Queen's Nurses' bags with a very ingenious arrangement for carrying bottles. He also shows some capital nurses' wallets, one made of aluminium especially took our fancy.

Southall Bros. and Barclay show their well-known "towels" and accouchement sets, as well as surgical and nursing instruments and appliances. They also show a "cremator" for the combustion of soiled "Sanitary Towels," a most desirable contrivance.

S. Maw, Son, and Thompson have a capital display, and among other ingenious devices we would refer to a hypodermic syringe without any packing, a glass tube, a glass plunger, a metal needle, and nothing else. Nothing could be more simple.

The Sanitary Wood Wool Company shows its dressings, pads, and accouchement sets, and other preparations, most of which have stood the test of many years.

From surgery we naturally turn to antiseptics, among the exhibitors of which we find A. Boake Roberts and Co. with their tubes of liquefied sulphurous acid gas; Arthur and Co., with antiseptic handkerchiefs; the "Sanitas" Company, with a fine display; Newton, Chambers, and Co., with Izal in many forms; Jeyes' Sanitary Compounds Company, with t eir well-established preparations; and other dealers in less well-known disinfectants.

Several stalls are occupied by medical publishers, who make a brave show of medical works. Rebman, with his fine "Blue-cover Series" and other works; John Bale, Sons, and Danielsson with their well-known charts and diagrams, together with a number of works on general medicine; and the Scientific Press, with a large show of medical, scientific, and nursing books, together with works on hospitals and other cognate subjects published by them, worthily represent the progress of medical science as depicted in medical literature.

Nor must we omit to mention Mrs. Ballin, with her magazines, "Womanhood" and "Baby," her baby clothes, and her various contrivances for making easy the art of rearing children.

Among the other objects of interest which were on view were the Dowsing Radiant Heat Apparatus and the filters shown by J. Defries and Sons and the Berkefeld Filter Company.

